# Understanding plant to extract ratios in botanical extracts

**DOI:** 10.3389/fphar.2022.981978

**Published:** 2022-09-30

**Authors:** Maria Monagas, Thomas Brendler, Josef Brinckmann, Steven Dentali, Stefan Gafner, Gabriel Giancaspro, Holly Johnson, James Kababick, Cuiying Ma, Hellen Oketch-Rabah, Pilar Pais, Nandu Sarma, Robin Marles

**Affiliations:** ^1^ United States Pharmacopeia (USP). Dietary Supplements and Herbal Medicines, Rockville, MD, United States; ^2^ United States Pharmacopeia (USP). Botanical Dietary Supplements and Herbal Medicines Expert Committee, Rockville, MD, United States

**Keywords:** botanical extract, genuine extract, native extract, plant to extract ratio, herbal medicine, drug to extract ratio (DER)

## Abstract

Dietary supplement current good manufacturing practice (cGMP) requires establishment of quality parameters for each component used in the manufacture of a dietary supplement to ensure that specifications for the identity, purity, strength, composition, and limits on contaminants are met.* Compliance with botanical extract ingredient specifications is assured by using scientifically valid methods of analysis, the results of which are reported on certificates of analysis (CoAs). However, CoAs routinely include additional data that are not amenable to verification through methods of analysis. Such descriptive information may include Plant to Extract ratios, which are ratios of the quantity of botanical article used in the manufacture of the extract to the quantity of extract obtained*.* Plant to Extract ratios can be misleading when their meaning is not clearly understood.

Plant to Extract ratios do not completely describe botanical extracts because other important factors influence the make-up of final extracts, such as the quality of the raw starting material (as can defined by pharmacopeial standards), extraction solvent(s) used, duration and temperature of extraction, and percentage and type of excipients present. Other important qualitative descriptions may include constituent “fingerprinting.” Despite these issues, Plant to Extract ratios are often used as a measure of extract strength for dosage calculations. This article defines and clarifies the meaning of Plant to Extract ratios and their proper use in describing and labeling botanical extract ingredients and finished products containing them.

## 1 Introduction

Botanical extracts are composed of extracted matter obtained from starting materials of botanical origin [United States Pharmacopeia (USP)]. They result from dissolving soluble plant constituents in extraction solvents and separating them from undissolved plant materials. A botanical extract has been defined as “the complex, multicomponent mixture obtained after using a solvent to dissolve components of the botanical material” (Dentali, 2013). Crude botanical extracts (those without added excipients) are called native or genuine extracts. Excipients are often added to extracts to improve their material handling characteristics, to standardize constituent concentrations, and for other functional purposes. The term “botanical extract” here refers to all types of extracts, independent of the relationship between identified constituents and the bioactivity (potency) of the extract.

Although botanical extracts may be subjected to additional processing to enrich the content of a particular chemical class of constituents, once they have been isolated as a fraction or as purified single constituents, these articles and expressed juices are not considered to be botanical extracts. Furthermore, extracts do not include chemically modified plant constituents except where artifacts result from heating or extraction processes. In addition, for the purposes of this article, the term plant or botanical is used in the broad sense to include algae, fungi, and lichens, as well as plant products such as exudates or oleo-gum-resins.

Botanical extracts can be described, in part, by the Plant to Extract ratio of botanical starting crude material (a.k.a. biomass) from which they were made, to the resulting native, or finished, extracts. The European Medicines Agency (EMA) [European Medicines Agency (EMA), 2010] guidelines refer to the ratio of starting material to genuine/native extract ratio as the DER genuine (Drug to Genuine Extract Ratio),^†^ and explain how to disclose the percent of genuine/native extract with the percent excipients for finished extracts. The DER is used as part of the strength characterization of an extract, i.e., the amount of starting material used to make a unit of extract. Community Herbal Monographs from EMA use the DER, together with extraction solvent information, for the determination of crude starting plant material equivalents in comparing extracts, and for estimating extract dosages according to well-established or traditional plant uses.

Plant to Extract ratios may enable the determination of an extract’s raw material equivalents, although the final chemical composition can vary depending on the quality of the starting plant material and the extraction conditions. A close comparability of extracts, also known as their phytoequivalence (Australian Government. Department of Health, 2011), cannot be determined without a detailed comparison of the solvent(s) and manufacturing processes used, sometimes supplemented with comprehensive comparisons of the extracts’ chemical compositions.

This article first provides an overview of botanical extracts, including their different types and forms, standardization, and categorization. Additional sections explain the concept of Plant to Extract ratios and the common misconceptions. Also provided are recommendations on how to apply the Plant to Extract ratio in labeling, and its relevance to the dosage of plant material equivalents.

## 2 Botanical extracts

Botanical extracts, as defined in the USP General Chapter <565> *Botanical Extracts* [United States Pharmacopeia (USP)]*,* are often used as ingredients in dietary supplements. Botanical extracts that conform to USP monographs for Botanical Extracts should be obtained from botanical articles that also conform to the corresponding USP monographs. Extracts may be manufactured to concentrate desired constituents, decrease the content of unwanted constituents or impurities, improve shelf life, and produce consistent material for the testing of claimed benefits. Depending on the type of botanical material and extraction technology used, prior to extraction the raw starting material may be subjected to different types of pretreatments, including cutting and grinding to reduce particle size and optimize surface area exposure, defatting, etc.

The composition of botanical extracts from the same plant may vary significantly depending on the extraction solvent(s) used, the temperature and duration of extraction, and the processes used to dry the extracts. Other sources of variation include the steps taken to concentrate or remove targeted constituents or classes of constituents, and the compounds formed during extraction or further processing. Additional variation in the composition of botanical extracts made using the same plant species and plant part as starting materials may occur due to genetic factors, environmental conditions, and agricultural practices. Managing the natural variations in starting material and using standardized extraction procedures can serve to create extracts with consistent composition. Suitable inert substances (excipients) are often added to extracts via granulation or other procedures to act as carriers or diluents which improve physical handling characteristics such as flowability and mixability. Excipients may also facilitate the production of a powder, reduce clumping, and improve homogeneity, bioavailability, stability, and other characteristics. Excipients can also be used to standardize the extract to a defined content of one or more constituents.

According to USP General Chapter <565> *Botanical Extracts* [United States Pharmacopeia (USP)], botanical extracts can be classified according to their physical state as either liquid (e.g., fluidextracts and tinctures), semisolid (soft), or solid (dry) forms. These physical forms are defined in Table 1.

**TABLE 1:** Botanical extract types and forms.

Extract type	Extract form	Definition	Examples of USP monographs
Fluidextract	Liquid	A type of liquid extract containing aqueous ethanol as a solvent or preservative, or both, made such that each 1 mL contains the extracted constituents of 1 g of the crude dry material that it represents, unless otherwise specified.	Black Cohosh Fluidextract
			Garlic Fluidextract
			Licorice Fluidextract
Tincture	Liquid	A type of liquid extract prepared with ethanol or hydroethanolic mixtures by maceration or percolation. Traditionally, tinctures of potent articles represent the activity of 1 g of the dry plant material in 10 mL of solvent, and in 5 mL for others.	*Rhodiola rosea* Tincture
			Valerian Tincture
Soft Extract & Oleoresins	Semi-solid or viscous	Extract articles with consistencies intermediate between those of liquid and dry extracts, with a consistency of a thin to thick liquid or paste. They either naturally have this consistency, as is sometimes obtained by extraction with supercritical carbon dioxide, or have it by virtue of incomplete evaporation of the extraction solvent and/or naturally occurring water content of the original biomass.	Capsicum Oleoresin
Dry Extract	Solid	Dry extracts are solid articles, available for example in powder, flake, granule, or other forms, obtained by evaporation of the solvent used in their production.	Guarana Seed Dry Extract
			Bitter Orange Fruit Flavonoids Dry Extract

## 3 Standardization of botanical extracts

Various organizations define botanical extract standardization differently

The American Herbal Products Association (AHPA) (American Herbal Products Association, 2003), the leading herbal trade organization in the United States, defines standardization as “the complete body of information and controls that serves to optimize the batch-to-batch consistency of a botanical product. Standardization is achieved by reducing the inherent variation of natural product composition through quality assurance practices applied to agricultural and manufacturing processes.” AHPA (American Herbal Products Association, 2021) points out that, “[i]n fact, standardization—when properly performed—entails a lot more than merely controlling the content of a particular marker compound … It comprises a wide variety of raw material and process controls, as well as use of a consistent recipe.”

Marker compounds are constituents that may or may not be associated with therapeutic activity and often are used as in-process controls. They also can help demonstrate identity when specific to the botanical raw material under consideration. On the other hand, marker compound levels may not vary proportionally with other compounds of greater importance relative to therapeutic activity, due to differences in genetics, growing conditions, or stability during processing and storage.

The EMA categorizes “standardised extracts” as those where the identified constituents are understood to fully account for an extract’s proven therapeutic activity [European Medicines Agency (EMA), 2010]. The relationship of identified constituents to an extract’s biological activity may not always be clear. Awang (2004) noted that the identity of constituents responsible for biological activities of a plant extract are rarely clearly established, even with bioassays and clinical studies, and numerous constituents may be active to different degrees and in various respects. A few examples of bioactive constituents in “standardised extracts” include the laxative sennosides of senna leaf, the hepatoprotective silymarin flavonolignans of milk thistle fruit, and the anti-nauseant gingerols of ginger rhizome.

Elimination of unwanted constituents, so-called negative markers, from extracts is also considered a form of standardization. Examples of negative markers include the neurotoxic thujones found in tansy, and hepatotoxic pyrrolizidine alkaloids found in comfrey and other herbs. Marker compounds include constituents that are characteristic of a particular species or variety of a plant, and thus are useful for standardization but may not be entirely responsible for the intended therapeutic activity. Examples include parthenolide in feverfew and echinacoside in *Echinacea angustifolia* and *E. pallida*.

Bioassays of extracts may provide some measure of therapeutic activity, although they are rarely used for standardization. A classic example is the use of *in vivo* bioassays with frogs, cats, and pigeons to standardize extracts of digitalis (Dieuaide et al., 1935; Lehman, 1936). In another example, bioassay is recommended as a way to ensure reproducible pharmacological activity (potency) of the dragon’s blood (*Croton lechleri*) latex botanical drug (U.S. Department of Health and Human Services, 2012). Although they may be useful for standardizing extracts to a potency measurement, challenges with bioassay-based standardization include cost, complexity, and demonstrating the relationship to clinically relevant effects in humans.

Standardization to either active or marker constituents and bioassays that reflect the underlying mechanisms of action were described by van Breemen et al. (2007). Hubbard et al. (2019) reported that recent developments in bioinformatics and bioassay technology have made it possible to address large numbers of phytochemical constituents in a plant extract and the potential diversity of their biological effects. This allows a much greater level of detail for characterizing the features that could be used for plant extract standardization.

## 4 Categorization of botanical extracts

In addition to the classification of extracts as to whether they are liquid, soft, or dry, the aforementioned EMA category of “standardised extracts” is joined by two other categories of extracts, namely “quantified extracts,” and “other extracts,” depending on the relationship between known constituents and the extract’s biological activity [European Medicines Agency (EMA), 2010]. Unlike the EMA-designated standardised extracts, in quantified extracts the identified constituents partly, but not fully, account for an extract’s bioactivity. In this case, and for “other extracts” that have no relationship between identified constituents and extract bioactivity, the whole of the extract is considered to be the active material.

Crude extracts may be processed further, or a selective extraction may be performed at the outset to concentrate particular classes of phytochemicals or to decrease the content of unwanted constituents, or both. Gymnema leaf extract containing NLT than 5.0% of gymnemic acids as *USP Purified Gymnema Extract*, and *USP Powdered Ginkgo Extract* that contains NLT 22.0% and NMT 27.0% flavonol glycosides, and NLT 5.4% and NMT 12.0% of terpene lactones, are examples of extracts in which specific constituents are enhanced. Licorice root deglycyrrhizinated extract and green tea leaf decaffeinated extract with a concentrated content of catechins as *USP Powdered Decaffeinated Green Tea Extract* represent extracts in which specific constituents have been removed.

**FIGURE 1:**
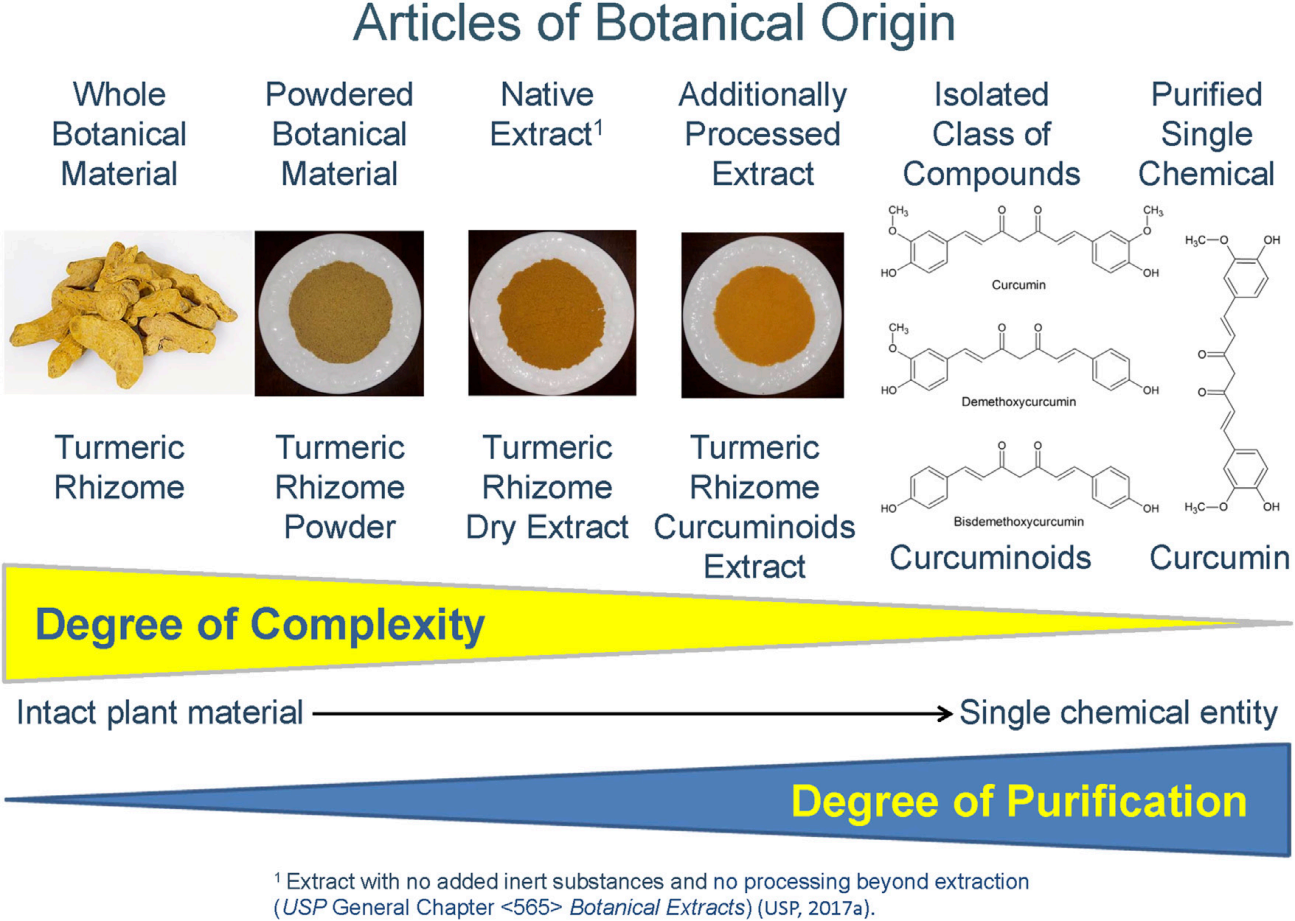
Article of Botanical Origin for Turmeric (*Curcuma longa* L.) rhizome form intact plant material to single chemical entity [United States Pharmacopeia (USP), 2019].

These specialized extracts fall along a chemical complexity continuum from selective and semi-purified botanical extracts or extract fractions that concentrate a phytochemical class, to an isolated class of phytochemical constituents, to a single compound (Figure 1). Depending on the degree of chemical purification, these latter two examples may no longer be appropriately considered extracts. Examples of concentrated constituent classes of compounds include sennosides extracted from senna leaflets or senna pods (*USP Sennosides*) and curcuminoids extracted from turmeric rhizome (*USP Curcuminoids*).

## 5 Plant to extract ratios: Definitions and misconceptions

It is important to clarify the concept of Plant to Extract ratios because misunderstandings regarding what they signify are common. Plant to Extract ratios reflect the amount of material extracted from plant biomass relative to the starting amount of biomass. They may be used to partly define extracts with or without the presence of added excipients. The calculations of Plant to Extract ratios should be made on the dried basis irrespective of whether the starting raw material used in the extraction is in fresh or dried form.^‡^

Similar to the EMA guidelines, the Kooperation Phytopharmaka (2016) defines the plant drug to extract ratio (DER) as the ratio of the amount of starting plant used to produce a certain amount of native extract that is exclusive of any carriers or other excipients. For example, a dry extract with an average native extract ratio of 10:1 means that approximately 10 g of dried raw material were required to produce 1 g of native/genuine extract.

In practice, native extract production yield will usually vary due to the inherent variation of extractive matter from different batches of starting materials; this results in a Plant to Extract ratio range in place of a single ratio. For example, using the same extraction conditions, one 100-kg lot of starting plant material may yield 14 kg of native extract while a different 100-kg lot may yield only 11 kg of extract. The Plant to Extract ratio in this case would be the range of 7 through 9 to 1, expressed as 7–9:1 (100 divided by 14 equals approximately 7, and 100 divided by 11 equals approximately 9). Only 7 kg of starting material would be needed to produce 1 kg of native extract in the first instance while 9 kg of another lot of the same plant would be required to produce an equivalent amount of extract.

Extract yields are fundamental to the calculation of Plant to Extract ratios. Perhaps the most common misconception regarding Plant to Extract ratios is that a higher ratio represents a stronger, and therefore better extract. Extract yields depend on the extraction process and the amount of extractable material in the starting plant biomass; Plant to Extract ratios describe the extract yield from a given raw material using a given manufacturing process. Consider a hypothetical case where all the starting material is converted to dry extract. In this case, the extract yield would be 100 percent and the Plant to Extract ratio would be 1:1,^§^ indicating that each unit of extract represents an equivalent amount of starting material. However, for most dry botanical materials extracted in aqueous or hydroethanolic solvents, the amount of extractable matter (soluble constituents) from the biomass is usually between 10 and 25 percent, which calculates to starting plant mass to dry extract ratios of 10:1 and 4:1, respectively (100 divided by 10 is 10, and 100 divided by 25 is 4).

The Australian Therapeutic Goods Administration (TGA) Guidance on Equivalence of Herbal Extracts in Complementary Medicines (Australian Government. Department of Health, 2011) states: “Whilst a high native extraction ratio is generally reflective of a targeted extraction procedure (i.e., specific components or component classes are selected for), there are instances where a high extraction ratio may simply reflect a partial extraction procedure.” For example, if a plant biomass with a potential 25% extractable crude material could have an extract ratio of 4:1 with a given manufacturing process but only yields 10% extractible materials of interest with a different process and leaves behind the other 15%, the extract ratio calculation changes from 4:1 to 10:1.

Low or high Plant to Extract ratios can be explained, in part, by the soluble extractive matter starting value. For example, woody roots may naturally contain relatively small amounts of extractable material and result in relatively high extract ratios even when extracted to exhaustion. According to the Hong Kong Chinese Materia Medica Standards, eleuthero root should contain not less than 3.0% water-soluble extractives and 3.0% ethanol-soluble extractives (using the cold extraction method in both cases) (Chinese Medicine Division and Department of Health, 2005a), thus a theoretical native extract ratio of about 33:1. In contrast, Asian ginseng root should contain not less than 27.0% water-soluble extractives and 22.0% ethanol-soluble extractives (using the cold extraction method in both cases) (Chinese Medicine Division and Department of Health, 2005b), thus a theoretical native extract ratio of about 4:1.

Another example of the challenge of using Plant to Extract ratios to compare botanical extract products on the market is illustrated by the case of Asian ginseng. The USP *Asian Ginseng Root and Rhizome* monograph sets out quality specifications for the dried roots and rhizomes of *Panax ginseng*, including minimum concentrations for ginsenosides Rg_1_, Re, Rf, Rb_1_, Rc, Rb_2_, and Rd. However, for dried raw material of a given age, the relative contents of ginsenosides Re, Rf, Rb_1_, Rc, Rb_2_, and Rd are significantly higher in the fibrous root portion > rhizome > branch root > main root, while the content of Rg_1_ is highest in the rhizome > branch root > fibrous root > main root (Pan et al., 2021). The rhizomes and main roots are often separated from smaller branch roots and fibrous roots in the material of commerce. Thus, a higher Plant to Extract Ratio would be needed using main root and rhizome material to achieve the same levels of the marker ginsenosides compared to extracts of the branch and fibrous roots.

As previously mentioned, high Plant to Extract ratios may also reflect manufacturing processes intentionally designed to capture only a narrow range of native constituents, either through use of a selective solvent for initial extraction or through further processing of crude extracts to concentrate specific constituents. For example, the *USP Native Gymnema Extract* monograph states that the ratio of starting plant material to extract is about 8:1. In contrast, *USP Purified Gymnema Extract*, prepared by further processing of *USP Native Gymnema Extract*, has a ratio of starting material to extract of about 25:1 because about two thirds of the native extract is discarded during preparation. Thus, in the case of the *USP Native Gymnema Extract*, it is evident that 100 g of starting material yields about 12.5 g of native extract, from which it is possible to create about 4 g of the purified extract (*USP Purified Gymnema Extract*).

Regarding extracts made from materials that yield high Plant to Extract ratios, the TGA makes this very important point: “Consideration should be given to ensuring that these extracts are not marketed in a manner that implies that they are “better” because they are derived from a larger quantity of raw herbal material. Such marketing would represent a misuse on the part of a supplier and a misunderstanding by customers” (Australian Government. Department of Health, 2011).

## 6 Plant to extract ratio and phytoequivalence

Botanical extracts are multi-component mixtures that can be produced to an acceptable consistency but are not usually completely uniform due to raw material variations and differences in manufacturing conditions. A full chemical comparison and/or biological testing may be needed to establish phytoequivalence between extracts so that the extracts can be assumed to be equivalent for all intents and purposes. Plant to Extract ratios that allow the calculation of starting material equivalents may serve as a criterion, along with other factors, to establish equivalence between different extracts (Health Canada, 2015). This applies only if sufficient manufacturing information about the finished extracts is available. This should include, at a minimum, the native extract concentration, extraction solvents used, and the general extraction procedure including steps applied to concentrate or remove constituents or classes of constituent (Australian Government. Department of Health, 2011). Ultimately, fingerprint characterization of constituents and quantification of marker or active compounds, as described in different sections of the USP botanical extract monographs, may be needed to fully establish phytoequivalence between extracts.

The Australian TGA Guidance on Equivalence of Herbal Extracts in Complementary Medicines (Australian Government. Department of Health, 2011) identifies the following as some of the factors that impact the phytoequivalence of extracts: starting material quality, solvent choices, and manufacturing processes including time and temperature. In relation to the solvent system, in cases where the type and amount of solvent used to manufacture a particular extract is the same, TGA states that a limited degree of variation in minor solvent concentration is now considered acceptable. In this way, extracts with small differences in extraction solvent systems may be considered phytoequivalent while excluding other solvent systems that could result in significant variation between extracts (Australian Government. Department of Health, 2011).

The addition of carriers and other excipients to extracts is another important aspect that should be addressed in the description of botanical extracts. According to the Australian TGA (Australian Government. Department of Health, 2011), “there are also situations where an extract with a high native extract ratio is diluted with a carrier or diluent, for a variety of purposes. The addition of diluents and carriers should always be taken into account when assessing whether two extracts are equivalent.”

The importance of differentiating between finished extracts containing excipients and 100% native extracts can be illustrated by considering finished extract ratios. For example, if an average of 4 kg of starting material is required to produce 1 kg of native extract, the average Plant to Extract ratio is 4:1. Adding 1 kg of carrier to each kg of native extract doubles the amount of total finished extract. Whereas the starting material to native extract ratio is still 4:1, the addition of carrier results in each kg of finished extract now containing 0.5 kg of native extract and 0.5 kg of excipient(s). The ratio of Plant to (finished) Extract (that is 50% native) is now 2:1. Without appropriate disclosure of the percentage of native extract or the percentage of excipients, a Plant to Extract ratio of 2:1 for this finished extract could imply a higher extraction yield than the original native extract ratio of 4:1. Therefore, accurate calculations of extract starting material equivalents require access to information regarding the percent of native extract and excipients in the finished extract.

## 7 Plant to extract ratio labeling

Plant to Extract ratio product labeling is required in some countries and not others, depending in part on the regulatory framework applicable for the finished product, i.e., whether the article is regulated as a food, a supplement, an over-the-counter (OTC) drug product, or a prescription drug product. This section covers ingredient labeling recommendations that are transferable to finished product labeling. Examples of Plant to Extract labeling guidelines from the Uniited States, Canada, and Australia are provided below.

### 7.1 Ingredients

In the United States, if a dietary supplement manufacturer claims that a dietary supplement ingredient meets USP standards, the product is misbranded (and thus unlawful) if it fails to actually meet those standards. The USP *Guideline for Assigning Titles to USP Dietary Supplement Monographs* (United States Pharmacopeia (USP), 2019) provides extensive details on the different types of botanical extracts for which monographs have been published, and how USP creates monograph titles that are part of the labeling requirements.

The USP General Chapter <565> *Botanical Extracts* (United States Pharmacopeia (USP)) in the *USP–NF* and in the USP *Dietary Supplements Compendium* states the following requirement for extract labeling: “Label it to indicate the name of the plant part used; the names of solvents, other than the hydroalcoholic solvents, used in preparation; the content, in percentage, of active principles or marker compounds identified in the individual monograph; and the name and concentration of any added antimicrobial or other preservative. Where active principles are unknown, the ratio of starting material to final product is stated. For semisolid extracts and powdered extracts, the identity and quantity of any added excipient is also indicated. In such cases, the percentage of native extract may also be stated.”

USP monographs for botanical extracts include Composition tests for percentage limits of identified active principles or marker compounds; manufacturers may also disclose both the extract ratio and excipient content. The EMA, in the 2010 *Guideline on Declaration of Herbal Substances and Herbal Preparations in Herbal Medicinal Products/Traditional Herbal Medicinal Products* (European Medicines Agency (EMA), 2010), recommends including the percent quantity of genuine extract, the DER (drug to genuine/native extract ratio) of the extract, and the percent amount of excipients in the declaration of ingredient descriptions, as can be seen in the following example:

Dry extract from Valerian root.

Quantity of the genuine extract: 80% genuine extract.

DER genuine: 3–6:1.

Other excipients: 20%

Extraction solvent: Ethanol 70% V/V.

### 7.2 Dosage forms

For dry (often referred to as “powdered”) extracts, Title 21 of the United States Code of Federal Regulations (21 CFR) section 101.36(b) (U.S. Department of Health and Human Services, 2012) (3) (ii) (C) states that “[f]or a dietary ingredient that is an extract from which the solvent has been removed, the weight of the ingredient shall be the weight of the dried extract.” 

In the case of liquid extracts, 21 CFR 101.36(b) [U.S. Department of Health and Human Services, 2012] (3) (ii) (B) states that for any dietary ingredient that is a liquid extract from which the solvent has not been removed, the quantity listed must be the volume or weight of the total extract. Information on the condition of the starting material must be stated when it is fresh and may be indicated when dried material was used to make the extract. Information may be included on the concentration of the dietary ingredient and the solvent used. The United States Food and Drug Administration (FDA) provides the following as an example: “fresh dandelion root extract, x (y:z) in 70% ethanol, where x is the number of mL or mg of the entire extract, y is the weight of the starting material, and z is the volume (mL) of solvent.” 

AHPA developed a retail labeling guidance for non-liquid botanical extracts titled *Guidance for the Retail Labeling of Dietary Supplements Containing Soft or Powdered Botanical Extracts* (American Herbal Products Association, 2000). This guidance includes carriers and other excipients as part of the quantity of a finished extract, which represents how bulk extracts are bought and sold–by total weight. AHPA also provides guidance on the voluntary disclosure of the percent of the native extract when it is listed on the label.

AHPA provides the following convention when manufacturers state extract ratios (American Herbal Products Association, 2000): “the first number shall represent the amount of dried botanical starting material, the second number shall represent the amount of *finished total extract* (emphasis added). For example, a 4:1 extract is one in which each kilogram (or other unit) of finished total extract represents the extractives from 4 kg (or other unit) of dried botanical starting material.” Following this convention, the amount of excipient is included in the calculation of starting material to finished extract.

AHPA offers two options for stating Plant to Extract ratios when lot-to-lot variation is encountered. These options are: 1) stating the range for either the native extract percent or for the extract ratio, or 2) using an average of the range when the range does not vary by more than 20% between the highest and lowest values (American Herbal Products Association, 2000). Two options also are provided for listing average extract values on a label, namely “average x% native” or “average x:1”. In practice, single values given for extract ratios generally represent a shorthand for the actual range. Additional information on extract ratios can be found in AHPA’s 2003 *White Paper: Standardization of Botanical Products* (American Herbal Products Association, 2003) and the 2000 *Guidance for the Retail Labeling of Dietary Supplements Containing Soft or Powdered Botanical Extracts* (American Herbal Products Association, 2000).

EMA (European Medicines Agency (EMA), 2010) guidelines provide detailed examples of how to declare Plant to Extract ratios that include disclosure of the percent excipients added to botanical extracts. The disclosure of excipients is directly translated to retail labeling of finished products. Following the earlier example of the Valerian root dry extract, Table 2 describes the correct labeling of a finished product (capsule) containing this ingredient.

**TABLE 2:** Labeling of a finished product (capsule) containing Valerian Dry Extract according to EMA (European Medicines Agency (EMA), 2010)

Ingredient	Finished Product (Capsule)
Dry extract from Valerian root	Quantity of the dry extract (genuine herbal preparation and other excipients) in the herbal medicinal product: 200 mg/capsule
Quantity of the genuine extract: 80% genuine extract	
DER genuine: 3–6: 1	Label Disclosure:
Other excipients: 20%	Each capsule contains 160 mg of extract (as dry extract) from Valeriana officinalis L s.l., radix
Extraction solvent: Ethanol 70% V/V	(Valerian root) (3–6: 1)
	Extraction solvent: Ethanol 70% V/V

The Natural and Non-prescription Health Products Directorate (NNHPD) in Canada specifies the listing of extract ratios on labels with the quantity of dried material used to make it, with the following as an example: Black Cohosh (6:1 extract) .... 40 mg, (*Actaea racemosa*) (root) equivalent to 240 mg of Black Cohosh (Health Canada, 2016). Since the amount of native extract or excipients is not specified, the extract ratio in this case takes into account the total amount of extract, including any added excipients, in order to represent the herb raw material equivalent.

*The Medicine Labels Guidance on TG O 91 and TG O 92, version 2.3* from the Australian TGA (Australian Government. Department of Health, 2021), states: “If the active ingredient in your medicine is a herbal preparation, its quantity must be expressed as the: weight of that preparation, and equivalent weight of the herbal material from which it was prepared.” Where “standardisation” is claimed (“the process in which the content of a specific chemical constituent(s) has been determined in a herbal material or herbal preparation”), “then the quantity of the active ingredient must be expressed as: the weight of that preparation, the minimum weight of the herbal material from which it was prepared, and the quantity of standardised constituent(s) in the herbal preparation.”

The *USP Asian Ginseng Root and Rhizome Dry Extract* example demonstrates the effect of the addition of excipients on both the native and final extract ratios. This article is prepared from the dried roots and rhizomes of *Panax ginseng* C.A. Mey. by extraction with water or hydroalcoholic mixtures. It contains not less than 3.0% of ginsenosides Rg_1_, Re, Rb_1_, Rc, Rb_2_, and Rd combined, calculated on the anhydrous basis, and may contain other added substances. If ten parts of starting material yields two parts of native extract, a 5:1 ratio of Plant to native Extract is obtained (10 divided by 2). If 0.5 part of excipient is added to the two parts of native extract, then the ratio of Plant to finished Extract becomes 4:1 (10 divided by 2.5 is 4). Best practices for labeling of the finished total extract ingredient according to USP General Chapter <565> *Botanical Extracts* [United States Pharmacopeia (USP)] and its corresponding communication in the finished product are as described in Table 3.

**TABLE 3:** Best practices for labeling of finished product (capsule) according to USP General Chapter <565> Botanical Extracts (United States Pharmacopeia (USP))

Ingredient	Finished Product (Capsule)
Plant to Extract ratio: 5:1	Quantity of finished total extract in the finished product: 250 mg per capsule
Excipients: 20%	
Solvents: Water or hydroalcoholic mixtures	Label Disclosure:
Content: 3.0% of ginsenosides Rg1, Re, Rb1, Rc, Rb2, and Rd combined, calculated on the anhydrous basis	Each capsule contains 200 mg of Asian Ginseng (Panax ginseng) Root and Rhizome Dry Extract corresponding to 6 mg of ginsenosides Rg1, Re, Rb1, Rc, Rb2, and Rd combined

## 8 Botanical extract ratio usage and comparison

Plant to Extract ratios provide an indication of strength relative to starting materials, including those recognized as traditional medicines. Plant to Extract ratios may be used to determine relevant raw material equivalents and form a reasonable basis for strength comparisons of raw materials and extracts. In fact, Health Canada states that: “an extract can be partially characterized by its specifications and the ratio of the quantity crude equivalent of the whole herb to the quantity of the extract” (Health Canada, 2015). EMA Community herbal monographs indicate the DER and solvent composition used for the manufacturing process; this DER is used to calculate the dose of the corresponding plant material (daily use) linked to the traditional use (European Medicines Agency, 2014). When active or marker compounds are known, it is recommended to include the quantity of constituents to confirm the relevant raw material equivalents.

Plant to Extract Ratios also play an important role in marketing authorization. Brand company applicants, whether seeking pre-marketing authorization for licensed Natural Health Products (NHPs) in Canada, listed Complementary Medicine Products (CMPs) in Australia, registered Remedios Herbolarios in Mexico, or registered Traditional Herbal Medicinal Products (THMPs) in the EU or UK, must declare in their quality dossiers the specified quality of each ingredient and the amount of excipients used. The Plant to Extract ratio is used in the efficacy dossier for determining dosage calculation. In most countries, Plant to Extract ratios are required to be disclosed on the label of the finished product.

## 9 Conclusion

This article explains the concept and use of Plant to Extract ratios, particularly with respect to dry extracts, and clarifies some of the common misconceptions regarding Plant to Extract ratios and their use. Plant to Extract ratios are important descriptors of botanical extracts linked to the extract yield of a manufacturing process. They may play a role in the estimation of phytoequivalence, labeling of botanical ingredients and corresponding dosage forms, and calculation of the plant material equivalents. To foster accurate communication between suppliers and manufacturers regarding botanical extracts, it is necessary to disclose not only the Plant to Extract ratios, but also to include the complete botanical extract composition, including any excipients and their percentage in the extract, extraction solvents, and the amount of active or marker constituents. In the absence of this information, the use of Plant to Extract ratios to calculate relevant raw materials should be considered with caution.

Plant to Extract ratios are among the descriptors of botanical extracts in the *Definition* and *Labeling* sections of USP monographs for botanical extracts. Manufacturers can follow the complete labeling recommendations for botanical extracts as described in <565> *Botanical Extracts,* which can help manufacturers to create accurate labeling of finished products containing botanical extract ingredients and support consumer decision-making when comparing similar products containing botanical extracts.

## Footnotes

† In this paper, the meaning of the term “Plant to Extract Ratio” is equivalent to “Drug to Extract Ratio (DER)” (used in Europe) and “Extract Ratio” (used in Canada and Australia). The term “herbal drug” as defined in the European Pharmacopoeia (also referred as “herbal substance” by the European Medicines Agency) is known as a *botanical dietary ingredient* as defined by the United States Food and Drug Administration (FDA), and hence the use of the term *Plant to Extract ratio* under the United States regulatory framework for Dietary Supplements.

‡ A dried botanical material is that which is in conformance with the Loss on Drying test in individual USP monographs.

§ In the case of liquid extracts, a 1:1 ratio defines fluidextracts.
